# From Fragmented Facts to Unified Knowledge: Exploring Concept Mapping in Neuromuscular Physiology Among First-Year Medical Students

**DOI:** 10.7759/cureus.74711

**Published:** 2024-11-28

**Authors:** Mangani Mangalavalli Shanmugarajah, Himel Mondal, Tina Das

**Affiliations:** 1 Physiology, All India Institute of Medical Sciences, Bhubaneswar, IND; 2 Physiology, All India Institute of Medical Sciences, Deoghar, IND; 3 Biochemistry, All India Institute of Medical Sciences, Bhubaneswar, IND

**Keywords:** active learning, competency-based medical education, concept mapping, critical thinking, general physiology, medical education, medical student, national medical commission, neuromuscular physiology, physiology

## Abstract

Background

First-year medical students may find it challenging to integrate complex physiological concepts, particularly neuromuscular physiology. While concept mapping has shown promise in medical education, its specific application in teaching intricate physiological mechanisms still needs to be explored. With this background, the objective of the study was to assess the feasibility of using concept mapping among first-year medical students and to explore the perception of students about concept mapping as an educational tool.

Methods

A mixed-methods study was conducted with first-year medical students (n = 110) of the 2023-2024 batch at All India Institute of Medical Sciences, Bhubaneswar, India. A briefing on the basic theories of concept mapping was carried out. Students participated in a structured concept mapping session focusing on the mechanism of muscle contraction. Students (two students in a group) created concept maps illustrating relationships between neuronal architecture, action potentials, neuromuscular transmission, sarcotubular system, excitation-contraction coupling, and muscle contraction processes. Data collection included digital submissions of concept maps and structured feedback questionnaires. Two faculty members evaluated the concept maps, and student feedback was analyzed using quantitative and qualitative approaches.

Results

A total of 110 first-year undergraduate medical students participated in the study and created 55 concept maps. The students scored 17.32 ± 1.7 out of 20 maximum achievable scores, which corresponds to an average of 86.59%. Seventy-five (68.18%) students rated the technique as excellent, 32 (29.09%) found it good, and only three (2.73%) rated it as average. Ninety-eight (88.8%) students strongly agreed or agreed that it provided a practical learning experience and found it refreshing compared to traditional lectures. Additionally, 102 (92.7%) students acknowledged its ability to stimulate creative thinking, and 102 (92.7%) also reported effective collaboration with peers. According to 82 (74.5%) students, concept mapping also facilitated in-depth knowledge acquisition, demonstrating its effectiveness in promoting active, engaging, and collaborative learning. Qualitative analysis revealed that concept mapping helped the students organize complex information, encourage critical thinking, improve retention through visual learning, promote collaborative knowledge-building, and facilitate self-assessment of their understanding.

Conclusion

Concept mapping can be used as a pedagogical tool for teaching complex neuromuscular physiology concepts to first-year medical students. The technique can bridge the gap between fragmented knowledge and integrated understanding while promoting active learning and critical thinking. The majority of the students rated it as excellent or good. It significantly enhances engagement, creative thinking, and deeper subject understanding compared to traditional lectures. Qualitative feedback underscores its role in improving comprehension of complex concepts and critical thinking skills.

## Introduction

Medical education faces an ongoing challenge: helping students transition from memorizing isolated facts to developing an integrated understanding of complex physiological processes. This challenge is particularly evident in neuromuscular physiology, where first-year medical students must grasp intricate relationships between neural control mechanisms and muscular responses. Traditional didactic approaches often fail to facilitate this crucial transition from fragmented knowledge to comprehensive understanding. To help with this, a visual learning strategy, concept mapping, was developed to enhance comprehension [1]. Concept mapping allows students to develop and integrate new ideas while facilitating the assessment of their learning capabilities [2].

Concept mapping, developed initially from Ausubel's theory of meaningful learning, offers a promising solution to this educational challenge [3]. Ausubel's pivotal distinction between rote memorization and meaningful learning in 1968 provided the theoretical foundation for concept mapping, which Novak later formalized as a structured approach to knowledge organization and integration [4-7]. This pedagogical tool enables learners to visualize and construct relationships between concepts, transforming abstract ideas into concrete, interconnected knowledge networks.

Concept mapping has emerged as a precious tool in medical education for several reasons. It aligns with the cognitive processes required for clinical reasoning, helping students to significantly strengthen their critical thinking and develop the mental frameworks necessary for evidence-based decision-making [5,8]. It serves as both a learning and an assessment tool, allowing educators to identify and address misconceptions early in the learning process [8]. In addition, it supports the development of critical thinking skills essential for medical practice by encouraging students to analyze and synthesize information actively [2,8].

Studies across various disciplines have shown that concept mapping yields positive outcomes when used as an instructional method [6,9]. Research indicates that concept mapping surpasses traditional teaching methods in facilitating knowledge transfer, comprehension, and information retention [10]. Concept mapping helps students visualize and connect intricate ideas, particularly in complex subjects like physiology, promoting deeper understanding rather than surface learning [11]. The maps are invaluable in medical education for reinforcing learned material, presenting new concepts, and assessing academic progress [12]. It can be utilized in clinical and lecture-based courses to gauge self-directed and experiential learning [13]. Furthermore, it has shown promise in supporting diverse learning needs, making it an inclusive pedagogical tool [14]. This approach enhances key competencies in medical training, such as critical thinking, knowledge synthesis, and deep understanding of complex concepts [15].

The application of concept mapping in neuromuscular physiology education represents a particularly compelling area for investigation. This topic's complexity, with its multiple interconnected systems and processes, makes it an ideal candidate for testing the effectiveness of concept mapping as a learning tool. While previous studies have explored concept mapping in various medical education contexts, a need to evaluate its specific impact on first-year medical students' understanding of neuromuscular physiology remains.

With this background, the objective of the study was to assess the feasibility of using concept mapping among first-year medical students and to explore the perception of students about concept mapping as an educational tool. By investigating these objectives, we aimed to contribute to the growing body of evidence supporting innovative pedagogical approaches in medical education and provide practical insights for educators teaching complex physiological concepts.

## Materials and methods

Study design and setting

This study was conducted with first-year MBBS students (n = 110) at All India Institute of Medical Sciences, Bhubaneswar, Odisha, India, during the 2023-2024 academic year. The study employed a mixed-methods approach, combining quantitative assessment of learning outcomes with qualitative analysis of student feedback.

Preparation phase

The research team first developed a structured framework for concept mapping, drawing on established principles and techniques from the literature. This involved creating detailed guidelines for students to construct their concept maps. Additionally, the team prepared examples of concept maps covering key aspects of neuromuscular physiology to serve as models during the implementation phase. Finally, assessment rubrics and student feedback instruments were carefully designed to evaluate the learning outcomes comprehensively. The concept mapping intervention was implemented following standard didactic lectures addressing the neurophysiological mechanisms of neuronal excitation and subsequent muscle contractile processes. Students were informed about the session on concept mapping and asked to prepare for the topic.

Implementation phase

The concept mapping intervention began with an initial briefing session, where the facilitators introduced the students to the fundamental principles and benefits of this active learning strategy. Randomly selected by a computer program, two students made a group. Students were then guided through a hands-on workshop (two hours duration) where they worked to create their concept maps illustrating the intricate mechanisms of neuromuscular physiology. Textbooks or notes were not allowed during the session. The topics covered included neuronal architecture, action potentials, motor unit recruitment, neuromuscular transmission, the sarcotubular system, excitation-contraction coupling, and the muscle contraction-relaxation cycle.

Throughout the mapping exercise, two faculty members provided ongoing support and guidance to the students, ensuring they could effectively translate their understanding of the subject matter into a visually coherent and conceptually robust representation.

Assessment phase

Upon completing their concept maps, each group of students was asked to photograph their work and upload the digital files to a designated Google Classroom platform, along with their names. Additionally, the participants (n = 110) individually filled out structured feedback questionnaires to share their experiences and perceptions. This questionnaire had a total of six closed-ended questions with Likert-type response options and one open question where students were free to share their opinions regarding the concept maps as a teaching-learning method.

**Figure 1:**
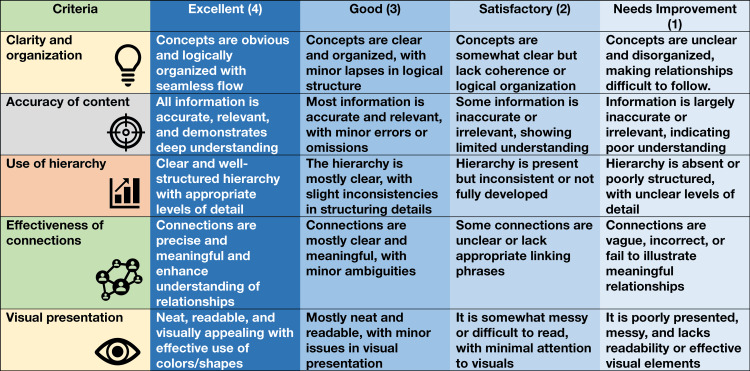
Rubrics for concept map assessment Dr Mangani Mangalavalli Shanmugarajah and Dr Tina Das developed the rubrics and Dr Himel Mondal visualized them and created the figure in Microsoft Word 2021 (Microsoft Corp., Redmond, WA, United States).

The evaluation process involved two independent faculty members assessing the submitted concept maps using the developed rubrics shown in Figure 1.

There were a total of five criteria, and each criterion had a maximum of four scores. Excellent concept maps for particular criteria (e.g., clarity and organization) were scored 4, and those that require improvement were scored 1. Two individual teachers scored the concept maps, and the average of their scores was taken as the final score.

Statistical analysis

Quantitative data was presented in numbers and percentages. The categorical data were compared statistically by the chi-square test, where a significant p-value indicates that the distribution did not occur by chance. We used GraphPad Prism 9.5.0 (Dotmatics, Boston, MA, United States) for statistical analysis. The qualitative data were thematically analyzed by QDA Miner Lite v.2.0.8 (Provalis Research, Montreal, Canada). A p < 0.05 was considered statistically significant.

## Results

A total of 110 first-year undergraduate medical students participated in the study. The students scored 17.32 ± 1.7 out of 20 maximum achievable scores, which corresponds to an average of 86.59%.

Seventy-five (68.18%) students rated the technique as excellent, 32 (29.09%) found it good, and only three (2.73%) rated it as average. The perceptions of the students are presented in Table 1.

**Table 1:** Perception of first-year medical students on concept mapping as an educational tool *p-value in a chi-square test.

Statements	Response, number (%)					p-value*
	Strongly agree	Agree	Neutral	Disagree	Strongly disagree	
Active learning by using concept mapping helped me gain a practical learning experience	49 (44.4)	49 (44.4)	8 (7.4)	4 (3.7)	0	<0.0001
Learning with concept mapping as a teaching-learning tool was refreshing compared to traditional lectures	65 (59.3)	45 (40.7)	0	0	0	<0.0001
Concept mapping stimulates creative thinking	86 (77.8)	16 (14.8)	8 (7.4)	0	0	<0.0001
Concept mapping enabled effective collaboration with peers	90 (81.5)	12 (11.1)	4 (3.7)	4 (3.7)	0	<0.0001
Concept mapping helped me acquire in-depth knowledge of the given topic	45 (40.7)	37 (33.3)	16 (14.8)	12 (11.1)	0	<0.0001

Ninety-eight (88.8%) students strongly agreed or agreed that it provided a practical learning experience and found it refreshing compared to traditional lectures. Additionally, 102 (92.7%) students acknowledged its ability to stimulate creative thinking, and 102 (92.7%) reported effective collaboration with peers. According to 82 (74.5%) students, concept mapping also facilitated in-depth knowledge acquisition, demonstrating its effectiveness in promoting active, engaging, and collaborative learning.

Qualitative analysis revealed that concept mapping helped students organize complex information, encourage critical thinking, improve retention through visual learning, promote collaborative knowledge-building, and facilitate self-assessment of their understanding. The themes and one direct quote of the students are shown in Table 2.

**Table 2:** Themes extracted from the thematic analysis of the open-ended question response provided by the students

Theme	Direct quotes from students
Organizes complex information	"It simplifies complex topics and helps me connect different concepts systematically"
Encourages critical thinking	"By creating links between concepts, I was able to think deeper about the 'why' and 'how' of each process"
Enhances retention through visualization	"The visual nature of concept maps makes it easier to recall details during exams"
Promotes collaboration	"Working in groups to create concept maps helped me learn from my peers and fill gaps in my understanding"
Assists in self-assessment	"When I create a concept map, I can clearly see what I don’t know and focus on those areas"

## Discussion

Our findings demonstrate that concept mapping can effectively bridge the gap between fragmented knowledge and integrated understanding in neuromuscular physiology. The visual representation of relationships, from neuronal activation to muscle contraction mechanics, enabled students to develop a holistic view rather than isolated pieces of information. The connection between ideas depicted by structured visualizations reinforces understanding and memory [6,7,9]. This aligns with Ausubel's theory of meaningful learning, emphasizing the superiority of integrated understanding over rote memorization [4,5].

This finding supports previous research that concept mapping encourages more profound engagement with learning material [15-18]. The visual nature of concept maps provides educators with valuable insights into students' thought processes, enabling targeted interventions for misconceptions and creating a feedback loop for supporting skills development [19]. One of the concept maps made by a student is shown in Appendix 1.

Medical education expects students to learn and integrate extensive information, for which student engagement is critical [10]. The positive student response suggests that concept mapping successfully addresses the engagement challenges and is an alternative to traditional lecture-based medical education [20,21]. This aligns with research showing active learning methods enhance student engagement [22,23]. The technique's effectiveness as a reinforcing tool in the undergraduate curriculum was particularly noteworthy [24]. Students' attitudes toward learning can improve if the learning methods are student-centric [25]. The positive feedback in concept mapping is contributed by its activity-based learning nature, which requires the student's active engagement in constructing their knowledge rather than passively receiving information.

We encountered several practical challenges during the implementation of the concept mapping intervention, which were proactively addressed. Ensuring adequate Wi-Fi connectivity was crucial to enable the digital submission of student concept maps in Google Classroom. Additionally, we provided a comprehensive orientation session to familiarize the students with the concept mapping techniques, as this was a novel learning strategy for many. A dual-evaluator system was implemented to maintain objectivity and consistency in the assessment process. Despite these logistical considerations, substantial benefits were observed in improving students' comprehension, critical thinking skills, and engagement with the complex subject of neuromuscular physiology.

Future research directions include investigating long-term knowledge retention, conducting comparative analyses with other visual learning tools, exploring applications in clinical reasoning development, and integrating concept mapping with digital learning platforms. By pursuing these avenues, educators can continue to build evidence-based strategies to optimize learning and prepare medical students for the demands of clinical practice.

The study was limited to a single cohort of first-year medical students at one institution. Additionally, the long-term retention of knowledge gained through concept mapping needed to be assessed, warranting further research to evaluate the scalability and sustainability of this approach across diverse educational settings and student populations. Although gender, cross-cultural, and intelligence biases were minimized through the random pairing of students, any persisting biases remained beyond our detection.

## Conclusions

This study demonstrates the feasibility of using concept mapping as a pedagogical tool in an Indian medical college for neuromuscular physiology. The technique successfully bridges the gap between fragmented knowledge and integrated understanding while promoting active learning and critical thinking. Students scored high grades in the formative assessment designed as a concept map. They perceived concept maps as a method that helps them gain practical learning experience with collaborative and creative learning that they thought was better than traditional learning methods. The high levels of student engagement, enhanced understanding, and development of critical thinking skills suggest that concept mapping can be a valuable addition to medical education curricula.

## Figures and Tables

**Figure 2:**
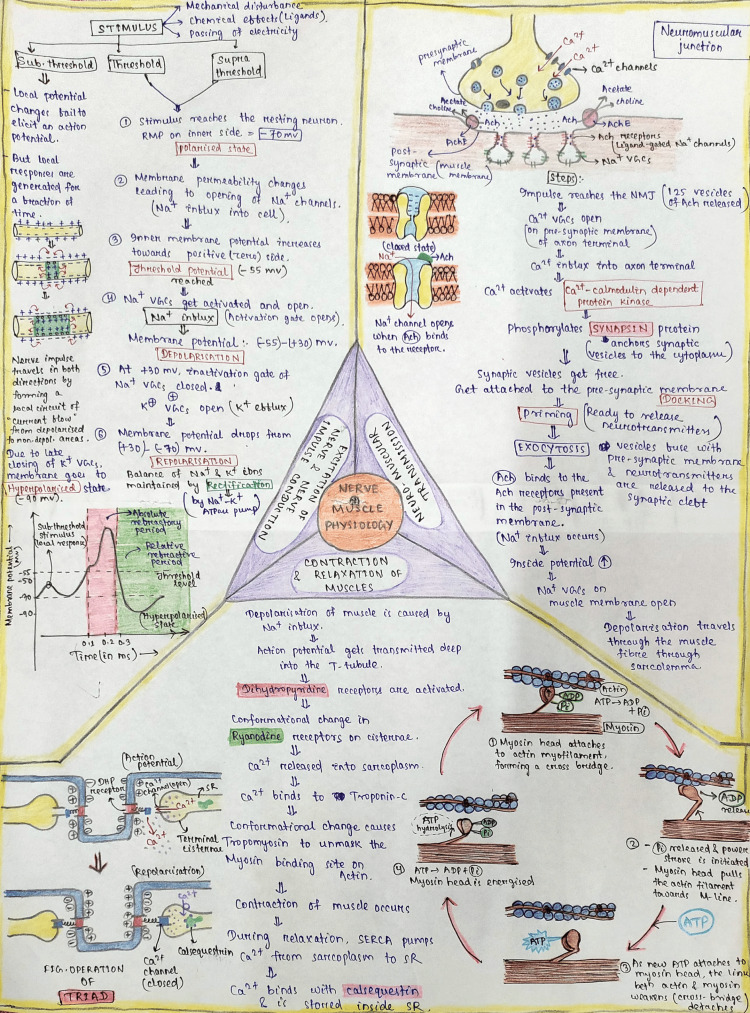
An example of a concept map created by a student during the session Permission was obtained from the student for the use of this concept map in this article.

## Appendices

Appendix 1 
